# Supplementary material for: Drosophila neprilysins control insulin signaling and food intake via cleavage of regulatory peptides
Source: eLife. 2016 Dec 6;5:e19430. doi: 10.7554/eLife.19430 (PMC5140268; doi:10.7554/eLife.19430)
Supplement: Figure 2—source data 1. — Significant changes are based on correlations with predictive scores from cross validated OPLS-DA models (Q2 = 0.95 and 0.74, respectively). A cutoff value for R2 corresponding to p<0.05 with Bonferroni correction for an assumed number of 100 metabolites was used. DOI: http://dx.doi.org/10.7554/eLife.19430.007 [file elife-19430-fig2-data1.docx]

| Metabolite | Chemical shifts of changing signals (ppm) | *mef2*-Gal4 x w1118 >  *mef2*-Gal4 x UAS-Nep4A | *mef2*-Gal4 x w1118 >  *mef2*-Gal4 x *nep4*-RNAi |
| --- | --- | --- | --- |
| NAD | 9.32, 9.13, 8.81, 8.42 | − | − |
| purine nucleotide | 8.52, 8.25, 6.13 |  | − |
| purine | 8.33, 8.22, 6.08 | + | + |
| histidine | 7.78, 7.05 | + |  |
| tyrosine | 7.18, 6.89 | − |  |
| trehalose | 5.18, 3.86, 3.83, 3.63, 3.44 | − |  |
| fructose | 4.10, 4.00, 3.88, 3.77, 3.68, 3.56 |  | + |
| glucose + fructose | 3.88, 3.77, 3.70, 3.57 | + |  |
| glutamine | 2.44, 2.12 | + | − |
| lactate | 1.32 | − |  |

**Figure 2-source data 1**

**Chemical shifts and detected changes of significantly affected metabolites.** Significant changes are based on correlations with predictive scores from cross validated OPLS-DA models (*Q*^2^=0.95 and 0.74, respectively). A cutoff value for *R*^2^ corresponding to p < 0.05 with Bonferroni correction for an assumed number of 100 metabolites was used.
